# Correction: The influence of FCGR2A and FCGR3A polymorphisms on the survival of patients with recurrent or metastatic squamous cell head and neck cancer treated with cetuximab

**DOI:** 10.1038/s41397-019-0073-5

**Published:** 2019-01-18

**Authors:** T. Magnes, T. Melchardt, C. Hufnagl, L. Weiss, C. Mittermair, D. Neureiter, E. Klieser, G. Rinnerthaler, S. Roesch, A. Gaggl, R. Greil, A. Egle

**Affiliations:** 1 Department of Internal Medicine III with Hematology, Medical Oncology, Hemostaseology, Infectious Disease, Rheumatology, Oncologic Center, Laboratory for Immunological and Molecular Cancer Research, Paracelsus Medical University, Salzburg, Austria; 2 Salzburg Cancer Research Institute (SCRI), Salzburg, Austria; 3 Cancer Cluster Salzburg (CCS), Salzburg, Austria; 4 Institute of Pathology, Paracelsus Medical University, Salzburg, Austria; 5 Department of Otorhinolaryngology, Head and Neck Surgery, Paracelsus Medical University Salzburg, Salzburg, Austria; 6 Department of Oral and Maxillofacial Surgery, Paracelsus Medical University Salzburg, Salzburg, Austria

Correction to: The Pharmacogenomics Journal;

10.1038/tpj.2017.37; published online 18 July 2017

In the abstract and in other parts of the manuscript the authors wrote that the mutation rs396991 causes a valine (V) to phenylalanine (F) substitution at position 157. However, the correct codon number is 158. These errors have not been fixed in the original article.

